# Flexible IZO/Ag/IZO/Ag multilayer electrode grown on a polyethylene terephthalate substrate using roll-to-roll sputtering

**DOI:** 10.1186/1556-276X-7-67

**Published:** 2012-01-05

**Authors:** Han-Ki Kim, Jong-Wook Lim

**Affiliations:** 1 Department of Advanced Materials Engineering for Information and Electronics, Kyung Hee University, 1 Seocheon-dong, Yongin-si, Gyeonggi-do, 446-701, South Korea

## Abstract

We investigated the optical, electrical, structural, and surface properties of roll-to-roll [R2R] sputter-grown flexible IZO/Ag/IZO/Ag [IAIA] multilayer films on polyethylene terephthalate substrates as a function of the top indium zinc oxide [IZO] thickness. It was found that the optical transmittance of the IAIA multilayer was significantly influenced by the top IZO layer thickness, which was grown on identical AIA multilayers. However, the sheet resistance of the IAIA multilayer was maintained between the range 5.01 to 5.1 Ω/square regardless of the top IZO thickness because the sheet resistance of the IAIA multilayer was mainly dependent on the thickness of the Ag layers. Notably, the optimized IAIA multilayer had a constant resistance change (Δ*R*/*R*_0_) under repeated outer bending tests with a radius of 10 mm. The mechanical integrity of the R2R-sputtered IAIA multilayer indicated that hybridization of an IZO and Ag metal layer is a promising flexible electrode scheme for the next-generation flexible optoelectronics.

## Introduction

Flexible transparent conducting oxide [FTCO] electrodes with superior flexibility have recently attracted much attention as key components in flexible displays, photovoltaics, and touch panels because device performances are critically dependent on optical, electrical, and mechanical properties of the FTCO [1-3]. In particular, the FTCO electrode should have mechanical robustness against substrate bending without resultant changes in its optical and electrical properties. Until now, amorphous indium tin oxide [ITO] electrodes have been widely employed as an FTCO material in flexible optoelectronic devices due to their high conductivity and transparency in the visible spectral range [4]. However, easy formation and propagation of cracks in the brittle amorphous ITO electrode have been considered as critical drawbacks of the amorphous ITO electrode in flexible optoelectronics [5]. To solve these critical drawbacks of the ITO electrode, flexible oxide-metal-oxide [OMO] multilayer electrodes, such as IZO/Ag/IZO, ITO/Ag/ITO, IZTO/Ag/IZTO, GZO/Ag/GZO, AZO/Ag/AZO, NTO/Ag/NTO, and ZTO/Ag/ZTO schemes, have been suggested due to their very low resistivity and high transparency [6-11]. Another promising candidate for FTCO is an inverted multilayer electrode, the metal-oxide-metal [MOM] multilayer structure. However, the existence of opaque metal layers could reduce the optical transparency of the MOM multilayer even though the MOM multilayer has a very low sheet resistance comparable to that of a metal electrode. Therefore, an additional thin oxide layer on the MOM multilayer is required to enhance the optical transparency of the MOM multilayer. However, the thickness effect of the top oxide layer on the electrical and optical properties of the MOM multilayer has not been investigated in detail.

In this work, we investigated the characteristics of flexible indium zinc oxide [IZO]/Ag/IZO/Ag multilayer electrodes grown on polyethylene terephthalate [PET] substrates using a specially designed roll-to-roll [R2R] sputtering system at room temperature. The electrical, optical, and mechanical properties of the IZO/Ag/IZO/Ag/PET samples were examined as a function of the top IZO thickness to decide the optimum thickness of the top IZO layer. In addition, surface morphology of the top IZO layer sputtered on the Ag/IZO/Ag multilayer was also investigated as a function of the top IZO thickness. Furthermore, mechanical stability of the optimized IZO/Ag/IZO/Ag multilayer under a repeated bending stress was examined using lab-made bending test system.

### Experimental detail

**Figure 1:**
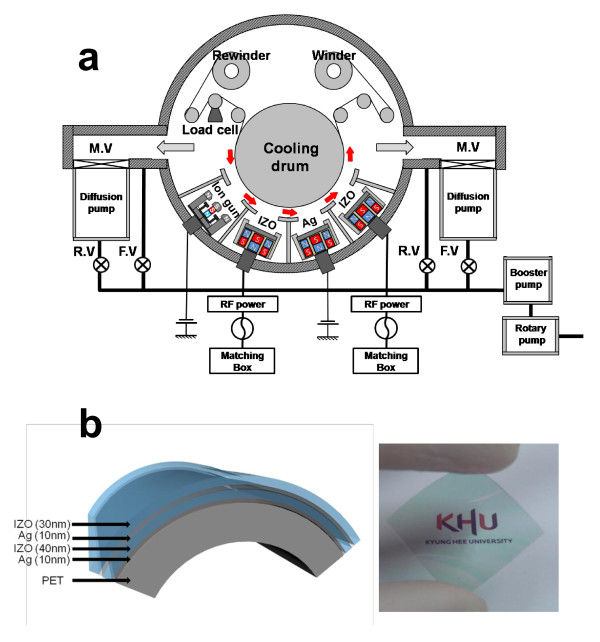
**R2R sputtering system schematic and IAIA sample structure**. (**a**) Schematic of a specially designed R2R sputtering system and (**b**) optimized structure of an IZO/Ag/IZO/Ag multilayer on a PET substrate and picture showing visual transparency of an IZO/Ag/IZO/Ag multilayer electrode.

The IZO/Ag/IZO/Ag multilayer electrodes were deposited on PET substrates using a specially designed lab-scale R2R sputtering system at room temperature. The R2R sputtering system equipped a rewind roller, an unwind roller, a cooling drum, three cathode guns, and a linear ion-beam source as shown Figure 1a. While 188-μm-thick PET substrates were passed over the main cooling drum, each layer was consecutively deposited on the PET substrate without substrate heating due to an excellent heat transport to the cooling drum. To ensure a good heat transport from the PET substrate to the cooling drum, an adjustable tension is applied to the rolling PET substrate by controlling the unwind and rewind rollers. An Ag (99.999% purity) metal target and an IZO (10 wt.% ZnO + 90 wt.% In_2_O_3_) ceramic target were placed at a distance of 100 mm from the main drum. Due to a limited number of targets, the IZO/Ag/IZO/Ag multilayer was prepared using two deposition steps. On the first step, the Ag (10 nm)/IZO (40 nm)/Ag (10 nm) multilayer was continuously deposited on the PET substrate at constant DC powers of 800 W for the Ag target and 350 W for the IZO target, an Ar flow rate of 30 sccm, a working pressure of 3 mTorr, and a rolling speed of 0.5 m/min. To restrict oxidation of the Ag metal target, reactive oxygen gas was not introduced into the chamber during the R2R sputtering process. Then, on the second step, the top IZO layer was deposited on the Ag/IZO/Ag multilayer as a function of thickness (0 to approximately 40 nm) at an identical sputtering condition with the bottom IZO layers. Thicknesses of the top IZO layers on the Ag/IZO/Ag multilayer were effectively controlled by the DC power of the IZO target. For simplicity, the IZO/Ag/IZO/Ag multilayer structure will be referred to hereafter as 'IAIA'. The thickness of each IZO and Ag layer was measured by a thickness profilometer (Surfcorder ET-3000, Kosaka Laboratory Ltd., Chiyoda-ku, Tokyo, Japan). A four-point probe (FPP-HS 8) and UV/Vis spectrometry (UV 540, Unicam) measurements were employed to investigate the effect of top IZO thickness on the electrical and optical properties of the IAIA electrode. Surface morphology of the top IZO layer and structure of the IAIA multilayer were analyzed by a field effect scanning electron microscope [FESEM] and X-ray diffraction [XRD] (D/MAX 2000, Rigaku Corporation, Tokyo, Japan), respectively. The mechanical flexibility of the optimized IAIA sample was analyzed using a lab-made bending test system [8]. The IAIA sample with a size of 1.5 × 7 cm^2 ^was mechanically clamped tightly between two 45° tilted Cu plates. To prevent increase of the resistance caused by the formation and propagation of cracks in the clamped IAIA region, we employed tilted Cu plates. By repeated linear motion of the Cu plates, we were able to flex and curve the IAIA sample and then measured the resistance change ((*R*_1_-*R*_0_)/*R*_0_) of the flexed IAIA sample simultaneously. The bending radius and frequency were approximately 10 mm and 60 Hz, respectively.

## Results and discussion

**Figure 2:**
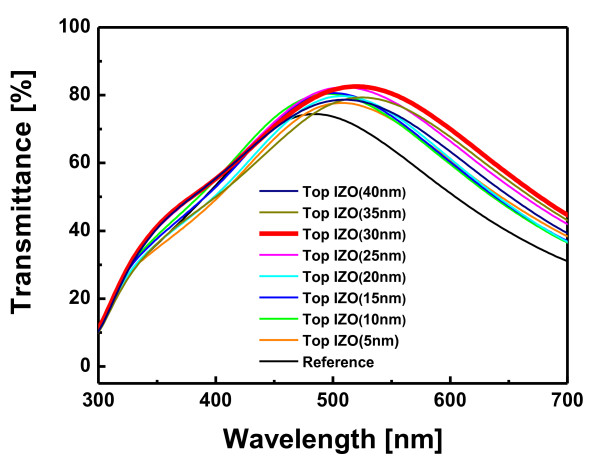
**Optical properties**. Optical transmittance spectra of IZO/Ag/IZO/Ag multilayer electrodes sputtered on PET substrates as a function of the top IZO thickness.

Figure 2 shows the optical transmittance of the R2R-grown IAIA multilayer electrodes on PET substrates as a function of the top IZO thickness. In the case of AIA (0 nm top IZO), it shows a low optical transmittance at specific wavelength regions of 300 to 400 nm and 600 to 700 nm due to the existence of metal Ag layers. The AIA multilayer electrode shows only a high transparency at 500 to 550 nm wavelength region, unlike that of the previously reported OMO multilayers [7-11]. This indicates that the antireflection effect is not observed in the AIA multilayer even though a very thin Ag layer (10 nm) was employed in the AIA structure. However, deposition of the top IZO layer on the AIA multilayer leads to an increase in the optical transmittance of the AIAI multilayer, especially in the wavelength above 500 nm. It was found that the optical transmittance of the IAIA multilayer increased with increasing top IZO thickness of up to 30 nm. The IAIA multilayer with 30-nm-thick top IZO layer showed the highest optical transmittance of 82.56%. This indicates that the 30-nm thickness of the top IZO layer is desirable to realize an antireflection effect in the AIAI multilayer structure. However, a further increase of the top IZO electrode thickness (35 nm and 40 nm) resulted in a decrease of transmittance. From these results, it is obvious that deposition of the top IZO layer on the AIA multilayer is effective to improve the optical properties of the inverted AIA multilayer electrode between 500 and approximately 700 nm wavelengths. However, to apply the AIAI electrode in optoelectronic devices, further improvement of optical transmittance in the blue wavelength region is necessary.

**Figure 3:**
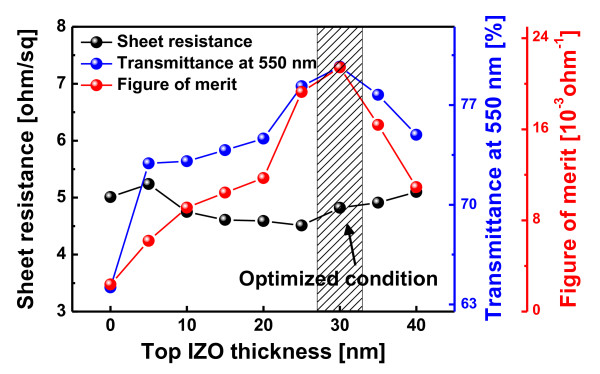
**Electrical properties**. Sheet resistance (*R*_sh_), optical transmittance at 550 nm, and figure of merit value of the IZO/Ag/IZO/Ag multilayer as a function of the top IZO layer.

Figure 3 shows the sheet resistance, optical transmittance at a wavelength of 550 nm, and figure of merit value (*T*^10^/*R*_sh_) calculated from the sheet resistance (*R*_sh_) and optical transmittance (*T*) of the AIAI multilayer electrodes as a function of the top IZO thickness. It was shown that the sheet resistance of the AIAI multilayer was fairly constant (approximately 5 Ω/square) regardless of the top IZO thickness due the Ag metal layer existence. The constant sheet resistance could be attributed to the existence of identical Ag layers in all IAIA multilayers. In our previous work, we reported that the sheet resistance and resistivity of the OMO multilayer were mainly determined by Ag thickness and morphology because most of the current in the OMO multilayer flowed through the inserted Ag metal layer [12]. However, the optical transmittance of the IAIA multilayer electrode at a wavelength of 550 nm mainly depended on the top IZO thickness. As expected from Figure 2, the IAIA multilayer with a 30-nm-thick top IZO layer showed the highest optical transmittance and figure of merit value. Because the sheet resistance of the IAIA multilayer electrode is constant regardless of the top IZO thickness, the figure of merit value also follows the optical transmittance. The figure of merit value also increases with the increasing top IZO thickness. A maximum figure of merit value of the IAIA multilayer could be obtained at a top IZO thickness of 30 nm as indicated by a shadow mark. It was thought that the IAIA multilayer showed a high optical transmittance and figure of merit value at 30 nm in thickness of the top IZO layer because the antireflection effect could occur at the symmetric oxide-metal-oxide [12]. However, a further increase of top IZO thickness leads to a decrease of the figure of merit value due to the diminishment of the antireflection effect in the IAIA multilayer.

**Figure 4:**
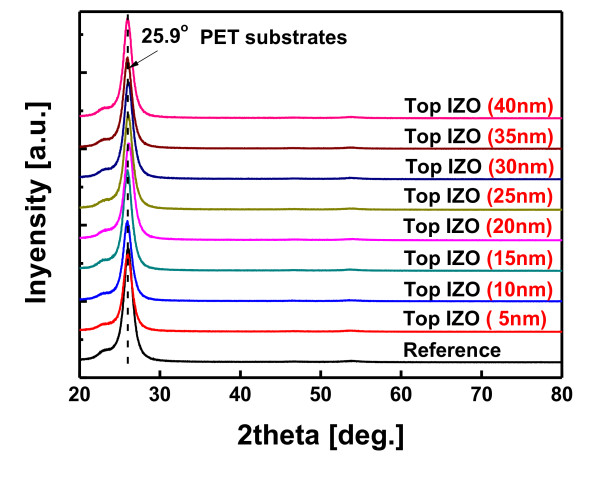
**Structural properties**. XRD plots of the IZO/Ag/IZO/Ag multilayer electrodes sputtered on PET substrates as a function of the top IZO thickness.

Figure 4 shows the XRD plots obtained from the R2R sputter-grown IAIA multilayer electrode as a function of the top IZO thickness. All XRD plots of the IAIA multilayer electrodes show only the intense PET substrate peak at the region of 25.84° regardless of the top IZO thickness. All XRD plots of the IAIA electrodes are identical. Due to a low PET substrate temperature during the R2R sputtering process, all IAIA multilayers exhibited a complete amorphous structure regardless of the top IZO thickness. However, it is difficult to examine the microstructure of the thin Ag layer (10 nm) in detail due to the limitation of our XRD power and resolution.

**Figure 5:**
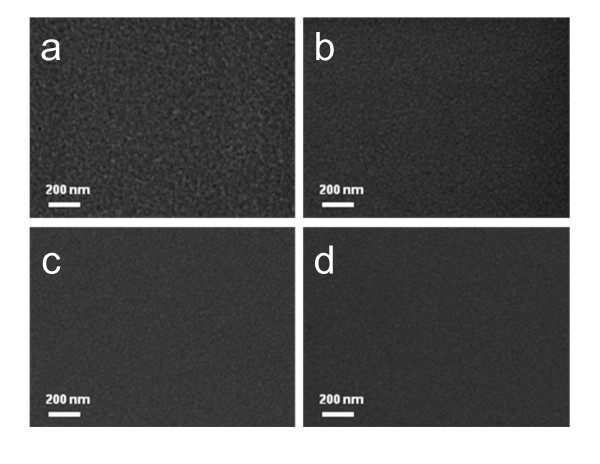
**Surface FESEM images**. Surface FESEM images of (**a**) reference Ag/IZO/Ag and (**b**) IZO (10 nm)/Ag/IZO/Ag, (**c**) IZO (20 nm)/Ag/IZO/Ag, (**d**) IZO (40 nm)/Ag/IZO/Ag multilayer electrodes.

Figure 5 shows the surface FESEM images of the Ag layer in the AIA multilayer and top IZO layer in the IAIA multilayer. In case of the 10-nm-thick Ag layer in Figure 5a, it shows randomly the connected network structure (island plus layer). However, the surface of the top IZO layer is fairly smooth and featureless without defect crack pinholes, cracks, and protrusion. In the case of the top IZO layer with a thickness of 10 nm in Figure 5b, it showed a surface image similar to that of the Ag layer in Figure 5a because it was directly sputtered on the randomly connected Ag islands. However, the surface of the top IZO layer gets smooth with the increased thickness because the island-like Ag layer was completely covered by the top IZO layer. At a top IZO thickness of 40 nm (Figure 5d), it shows a very smooth surface morphology.

**Figure 6:**
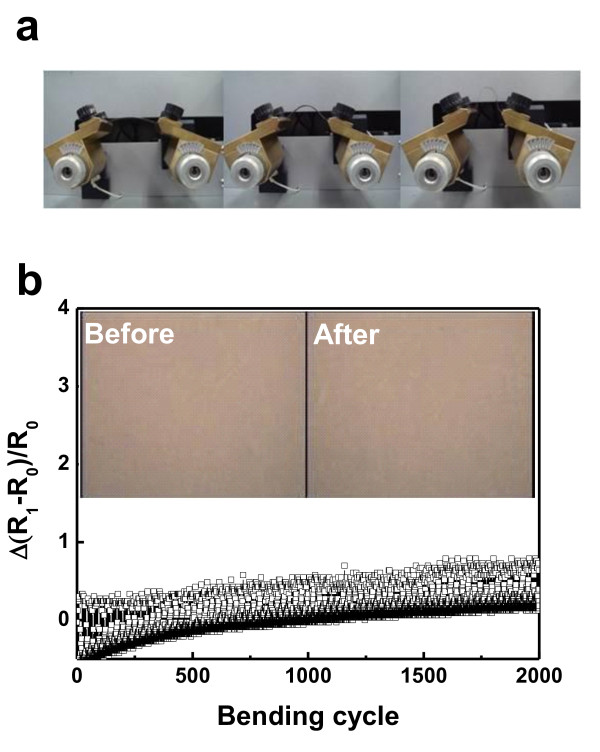
**Bending test and *in-situ *measured resistance**. (**a**) The pictures of the bending test steps and (**b**) variation of the *in-situ *measured resistance ((*R*_1_-*R*_0_)/*R*_0_) during repeated bending cycles for R2R-sputtered IZO/Ag/IZO/Ag multilayer electrodes, with inset optical microscope images of the top IZO layer before and after the bending test.

Figure 6a showed the pictures of the outer bending test using a lab-made bending test system. Unlike conventional bending test systems, our system employed a tilted Cu clamp in order to avoid the formation of cracks in the Cu-clamped region. Figure 6b exhibited the change of measured resistance from the IAIA multilayer with inset of the optical microscope images obtained from the top IZO layer before and after the bending test. Changes in the resistance of the flexible IAIA multilayer electrode (IZO (30 nm)/Ag (10 nm)/IZO (40 nm)/Ag (10 nm) sample) were expressed as (*R*_1_-*R*_0_)/*R*_0_, where *R*_0 _is the initial resistance and *R*_1 _is the measured resistance value after substrate bending. The optimized IAIA multilayer electrodes exhibited a constant resistance throughout the 2,000 bending cycles. The constant OM images before and after the bending test indicate the robustness of the IAIA multilayer electrode. The superior flexibility of the IAIA multilayer electrode could be attributed to the existence of an Ag metal layer between the IZO layers. Lewis et al. reported that the presence of an Ag layer between the ITO layers provides effective electrical conductivity even after the ITO is stressed beyond its failure strain (approximately 0.8%) due to the higher failure strain of the bulk-like Ag film (4% to approximately 50%) [13].

## Conclusions

In summary, we investigated the characteristics of IAIA multilayer electrodes grown on PET substrates using a specially designed lab-scale R2R sputtering system at room temperature. In two steps, we prepared the IAIA multilayer as a function of the top IZO thickness. It was found that the optical transmittance of the IAIA multilayer was significantly influenced by the thickness of the top IZO layer, which was grown on identical AIA multilayers. However, the sheet resistance of the IAIA multilayer was maintained between the range 5.01 to 5.10 Ω/square regardless of the top IZO thickness because the sheet resistance of the IAIA multilayer was mainly dependent on thickness of the Ag layers. In addition, all IAIA multilayers show an amorphous structure and a very smooth surface due to the low substrate temperature which is maintained by the cooling drum. Furthermore, the high failure strain of the inserted Ag layer improved the robustness of the IAIA multilayer electrode. These results indicated that the R2R sputter-grown IAIA multilayer electrode is a promising candidate to replace conventional amorphous ITO electrodes.

## Competing interests

The authors declare that they have no competing interests.

## Authors' contributions

JWL carried out the R2R sputtering process and measured electrical and optical properties of the IZO/Ag/IZO/Ag multilayer electrodes. HKK designed the experiments and wrote the manuscript. All authors read and approved the final manuscript.
